# Dual S-methoprene and *Lysinibacillus sphaericus* larvicide use leads to multiple independent, and not cross-resistance in *Culex pipiens*

**DOI:** 10.1371/journal.pone.0332621

**Published:** 2025-09-29

**Authors:** Kristina Lopez, Patrick Irwin, Mark Tomek, Robert Holub, Susan Paskewitz, Lyric Bartholomay, Mark Clifton

**Affiliations:** 1 North Shore Mosquito Abatement District, Northfield, Illinois, United States of America; 2 Northwest Mosquito Abatement District, Wheeling, Illinois, United States of America; 3 Department of Entomology, University of Wisconsin-Madison, Madison, Wisconsin, United States of America; 4 Desplaines Valley Mosquito Abatement District, Lyons, Illinois, United States of America; 5 Department of Pathobiological Sciences, University of Wisconsin-Madison, Madison, Wisconsin, United States of America; Benha University Faculty of Veterinary Medicine, EGYPT

## Abstract

S-methoprene, an insect growth regulator, and *Lysinibacillus sphaericus* (*Ls*), an entomopathogenic bacterium, are important larvicides used to control *Culex pipiens* [L.] mosquitoes, the primary vector of West Nile virus, in the Chicago, IL USA region. Resistance to both agents has been documented globally including a report of resistance ratios greater than 100 to S-methoprene in the Chicago region. Laboratory studies have suggested the potential for unidirectional cross-resistance between S-methoprene and *Ls*, despite differing modes of action. Among wild populations of *Cx. pipiens* in the Chicago area, this study aimed 1) to assess resistance status to *Ls*, 2) confirm the presence of S-methoprene resistance ratios >100, 3) determine if higher S-methoprene resistance ratios are associated with higher *Ls* resistance ratios, or whether *Ls* resistance arises solely from *Ls* exposure, and (4) determine the relationship between *Ls* treatment history and resistance levels of that active ingredient. We assessed susceptibility to both S-methoprene and *Ls* in 32 *Cx. pipiens* populations: 19 with S-methoprene exposure but no *Ls* history, and 13 with multi-year exposure to both larvicide active ingredients. *Ls* susceptibility was evaluated using dose-response bioassays to estimate LC_50_, LC_90_, and resistance ratios. Susceptibility to S-methoprene was tested using diagnostic doses corresponding to resistance ratios of 10 and 100 at the LC_50_. Resistance ratios to S-methoprene exceeding 10 were detected in 30 of 32 sampled populations. Among the 13 sites with prior *Ls* exposure, 11 were observed with resistance ratios > 5. In contrast, none of the 19 populations without *Ls* exposure exhibited *Ls* resistance, despite exhibiting higher S-methoprene resistance ratios. This lack of overlap supports the conclusion that S-methoprene resistance does not confer cross-resistance to *Ls* in the studied region. Logistic regression revealed a strong association between *Ls* treatment history and resistance development. The probability of *Ls* resistance exceeded 80% after 10 *Ls* applications within an eight-year period. These findings emphasize the need to develop improved resistance management strategies for larvicidal insecticides.

## Introduction

Mosquito-borne diseases pose an increasing global public health threat [1], particularly for pathogens for which no effective vaccines or prophylactic treatments exist such as West Nile virus (WNV) [2]. In the absence of broadly available medical interventions, vector control remains the primary strategy for reducing mosquito populations, interrupting pathogen transmission, and preventing disease outbreaks [3,4]. Integrated Mosquito Management (IMM) programs are recommended to employ a combination of complementary approaches, including public education, larval habitat reduction, vector and pathogen surveillance, action thresholds for insecticidal treatment, and the use of insecticides to control adult and larval mosquitoes [5,6]. Mosquito Control Organizations (MCOs) in the Chicago, IL, USA region typically employ the full range of IMM approaches, including larvicidal insecticides, to mitigate the threat posed to human health by seasonal outbreaks of WNV.

Larvicidal insecticides such as the insect growth regulator S-methoprene, and the entomopathogenic bacterium *Lysinibacillus sphaericus* [Meyer and Neide] (*Ls*), are extremely valuable tools in mosquito control due to their targeted use-pattern, favorable environmental profile, high specificity for mosquitoes, diverse and user-friendly formulations, and potential for extended residual activity [7,8]. Because of these advantages, these materials have enjoyed widespread adoption within mosquito control programs in the Chicago region where they are used to reduce the populations of the primary vector of WNV, *Culex pipiens* [L.]. However, prolonged and widespread use of larvicides, like other insecticidal interventions, can lead to resistance in exposed mosquito populations, ultimately threatening the effectiveness of control programs and their ability to reduce vector populations and prevent disease [9,10]. To mitigate the risk of resistance, the rotation of insecticidal active ingredients with different modes of action is widely recommended to reduce sustained selective pressure from a single active ingredient [3,5,7–12]. However, in an operational mosquito control context, limited guidance exists on key aspects of rotational strategies, including the optimal sequence of products, timing of rotations, geographic considerations, and the potential for cross-resistance between active ingredients that could negate rotational schemes [3,5].

Resistance to S-methoprene and *Ls* have been periodically reported for members of the *Culex* species complex (*Cx. pipiens* and *Culex quinquefasciatus* [Say]). Resistance to *Ls* in *Cx. quinquefasciatus* has been reported from a diverse range of global locations such as France, Brazil, India, China, and Tunisia [reviewed in 7], as well as in *Cx. pipiens* from multiple locations within the United States such as California [13] and Utah [14]. S-methoprene resistance in *Cx. pipiens* has been much less commonly reported globally with only a handful of reports of “low” to “moderate” resistance from the United States [15,16]. Resistance ratios (RRs) exceeding 100 to S-methoprene were recently documented in *Cx. pipiens* from Chicago, IL, and were described by the authors as indicative of extreme resistance [17], highlighting the urgent need for effective rotational and other resistance management strategies in the region.

Despite growing evidence of resistance to both S-methoprene and *Ls* in various *Culex* spp. populations, few studies have examined whether selective pressure by one of these active ingredients might confer resistance to the other. This question is particularly relevant given the reliance on product rotation strategies in operational control programs, where cross-resistance between active ingredients could undermine the effectiveness of such approaches.

Notably, field collected *Cx. pipiens* populations from both Chico, California [13] and Salt Lake City, Utah [14] were highly resistant to *Ls* yet remained completely susceptible to S-methoprene. In the few examples of S-methoprene resistance that have been published with field-collected *Cx. pipiens*, *Ls* resistance was not concurrently assessed [15–18]. The potential for unidirectional cross-resistance between S-methoprene and *Ls* has only been observed through laboratory selection experiments [19]. In this prior study, field-collected *Cx. quinquefasciatus* were artificially selected in the laboratory for high-levels of S-methoprene resistance (resistance ratio ~168) which resulted in a ~ 77-fold increase in resistance to *Ls*, despite no direct exposure to *Ls* or deliberate selection for *Ls* tolerance [19]. These findings suggest the possible existence of an unknown unidirectional cross-resistance mechanism between S-methoprene and *Ls* in *Culex* spp. [19]. When this laboratory-derived result is considered contextually with prior observations of S-methoprene resistance in Chicago-area populations of *Cx. pipiens* [17]*,* it suggests that *Ls* resistance may exist in locations with no *Ls* treatment history due solely to the presence of S-methoprene resistance. Such an outcome would critically undermine the presumed independence of these active ingredients in rotational schemes. 

This issue must be viewed in the operational context of mosquito control, where limited options for active ingredients and logistical constraints contrast sharply with the broader chemical and management options available in agriculture. Rotation is often the only feasible resistance management tactic, making it especially vulnerable if cross-resistance between larvicides goes undetected. Monitoring for cross-resistance is thus inseparable from evaluating the overall success of rotation strategies. Confirmation of cross-resistance between S-methoprene and *Ls* in natural *Cx. pipiens* populations would fundamentally challenge the viability of current rotational strategies and expose a critical vulnerability in mosquito control’s limited resistance management toolkit.

To address the potential for a unidirectional cross-resistant relationship between S-methoprene and *Ls*, this study aimed to address four related questions: (1) Does *Ls* resistance exist in the *Cx. pipiens* populations from the Chicago, IL region? (2) Do high levels of S-methoprene resistance also exist in these mosquito populations? (3) Does the existence of S-methoprene resistance lead to *Ls* cross-resistance in local mosquito populations, or alternatively, is *Ls* resistance strictly a result of direct *Ls* exposure? And (4) what is the relationship between *Ls* treatment history and the development of *Ls* resistance? To answer these questions, we evaluated 32 *Cx. pipiens* populations collected from three mosquito control districts in the Chicago area, all with a long-term history of S-methoprene use but varying histories of *Ls* use. We hypothesized that due to the S-methoprene resistance previously identified across the region [17], and because S-methoprene selection led to *Ls* cross-resistance in artificial selection experiments [19], areas without a *Ls* treatment history (but with RRs > 100 for S-methoprene) would also demonstrate *Ls* resistance despite a lack of exposure. By comparing susceptibility patterns of these two active ingredients between populations with different treatment histories, we ultimately aimed to determine whether the development of S-methoprene resistance precludes the use of *Ls* as a rotational tool in resistance management strategies.

## Methods

### Study sites

**Fig 1:**
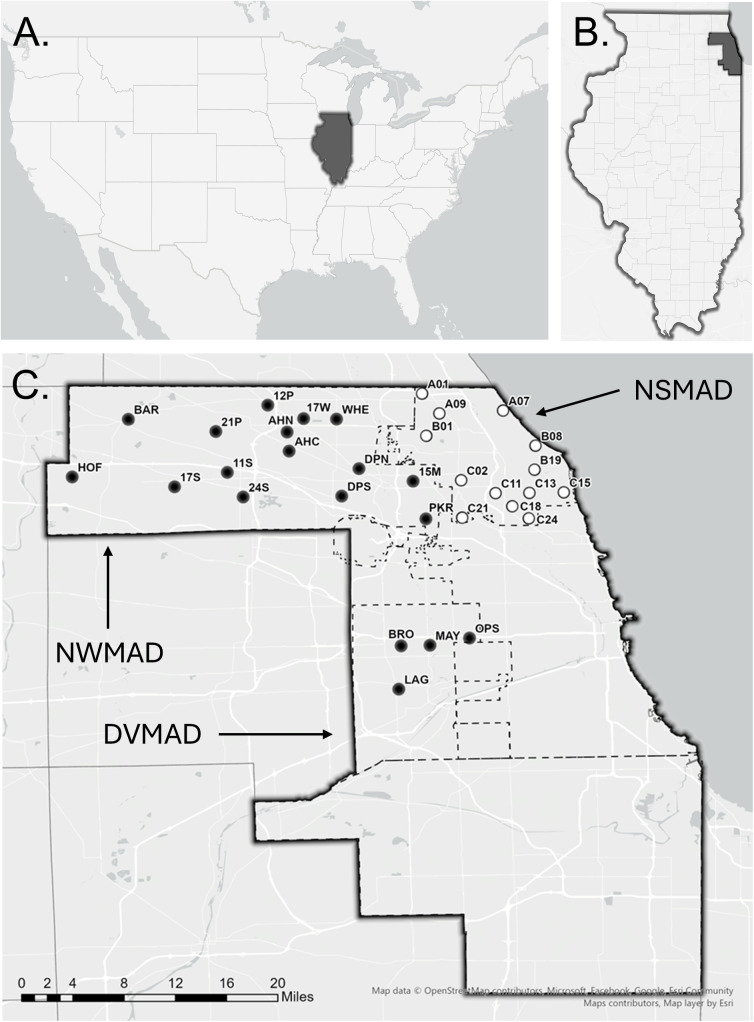
Map of study area. **(A)** Map of the United States of America highlighting Illinois, **(B)** Map of Illinois highlighting Cook County, **(C)** Study sites within Cook County. All sample locations have an S-methoprene treatment history. Unfilled circles indicate populations from sites with *Ls* treatment history, where black filled circles indicate populations without any *Ls* treatment history. Map created by Austin Robak using Esri basemap data © OpenStreetMap contributors, Esri, under CC BY 4.0 (https://creativecommons.org/licenses/by/4.0/).

*Culex pipiens* egg rafts were collected from 32 sites across three MCOs in the northern and western suburbs of Chicago, IL (Fig 1; S1 Table). Each sample site encompassed approximately 1.3 km² and was separated from other sample sites by at least 1.6 km. All 32 sample sites have a long-term history (>10 years) of S-methoprene use, primarily applied to urban and suburban stormwater catch basins. Collection sites from the Northwest Mosquito Abatement District (NWMAD; no history of *Ls* use) included Barrington (BAR), Wheeling (WHE), Hoffman Estates (HOF, 17S), Arlington Heights (AHC, AHN, 17W), Des Plaines (DPS, DPN), Park Ridge (PKR, 15M), Schaumburg (11S, 24S), and Palatine (12P, 21P). Sites from the Desplaines Valley Mosquito Abatement District (DVMAD; no history of *Ls* use), included Broadview (BRO), La Grange (LAG), Maywood (MAY), and Oak Park (OPS).

In contrast, the North Shore Mosquito Abatement District (NSMAD) has a > 10 year history of S-methoprene applications and introduced *Ls* larvicide applications to stormwater catch basins in 2016 as a rotational strategy. Collection sites from this district include Glencoe (A07), Wilmette (B08), Glenview (B01), Morton Grove (C02), Niles (C21), Lincolnwood (C24), Northbrook (A01, A09), Evanston (B19, C15), and Skokie (C11, C13, C18). A susceptible laboratory strain of *Cx. pipiens* (COL), originating from Iowa and maintained at the University of Wisconsin–Madison, was used as a reference. The Iowa strain is fully susceptible to pyrethroids and insect growth regulators and has no known exposure history to either S-methoprene or *Ls* [17,20].

No permits were required for this study. Field work was conducted by staff of the NSMAD and cooperating personnel from NWMAD and DVMAD with access granted through internal operational authority and verbal agreement between participating districts.

### Mosquito collection and rearing

Egg rafts were collected from two to four gravid ovitraps per site, each baited with an alfalfa pellet infusion. Collections were conducted between July and September 2024 (epidemiological weeks 29–39). Each of the 32 sites was sampled one to three times, with sequential-day sampling used for sites visited multiple times. A minimum of 8 egg rafts was required for a sample location to be included. After collection, individual egg rafts were placed in 6 oz. Styrofoam cups (Item 6SJ12, Dart Container Company, Mason, MI, USA) containing ~150 mL of tap water.

Second instar *Cx. pipiens* larvae were identified morphologically [21] and fed ground TetraMin® tropical fish flakes until assignment to a larvicide bioassay (Spectrum Pet Brands LLC, Blacksburg, VA, USA). Other *Culex* spp. larvae were excluded from the experiment. To minimize interfamilial, temporal, or generational effects from egg raft collection over two or three days, identified *Cx. pipiens* larvae from different ovitraps within the same site were pooled and reared under standardized conditions (27°C, 80% RH, 16:8 light:dark cycle) until they reached the appropriate developmental stage for bioassay evaluation.

### Larvicide bioassays and probit analysis

From each pooled sample population, larvae were randomly assigned to one of two bioassay types: a dose-response bioassay for *Ls* or a diagnostic-dose bioassay for S-methoprene. This design allowed simultaneous evaluation of resistance to both larvicides using larvae from the same field population. The use of a diagnostic-dose for S-methoprene greatly reduces the number of larvae required to assess resistance to this material. Approximately 70% of reared larvae were used in *Ls* bioassays and 30% in S-methoprene bioassays.

To assess the presence and intensity of *Ls* resistance, we conducted dose-response bioassays followed by probit analysis to estimate LC_50_ and LC_90_ values, determine 95% confidence intervals, and calculate RRs. Bioassays followed previously described protocols [13,14,22]. In brief, approximately 25 third-instar *Cx. pipiens* larvae were placed in 6 oz Styrofoam cups containing 100 mL of tap water. To remain consistent with the feeding regimen and bioassay design outlined in [13,14], and [22] ~100 mg of rabbit pellets were added to the Styrofoam cups (Kaytee Products Inc., Clinton, WI, USA) as food. VectoLex WDG (51.2% *Lysinibacillus sphaericus* 2362, Serotype H5a5b, strain ABTS 1743; Valent BioSciences, Libertyville, IL, USA) was serially diluted with tap water to prepare 8 stock solutions ranging in concentration from 1 x 10^7^ ppb (1% w:v) to 1 ppb (1 x 10^−7^% w:v). Stock solutions were stored at 4°C in borosilicate amber glass bottles and acclimated to room temperature before use. Stock solutions were discarded and re-prepared every three days to maintain potency (personal communication, Tianyun Su). Bioassay cups were covered with modified dome-shaped clear plastic lids [17] and maintained under ambient laboratory conditions (23°C, 60% RH) in a separate laboratory to avoid contaminating larval rearing facilities.

Each field-collected population and the susceptible laboratory strain (COL) were evaluated using a minimum of three replicates for each *Ls* concentration tested. When sufficient larvae were available, additional replicates and/or concentrations were conducted to improve probit model fit, resulting in 3–6 replicates per population across 10–21 concentrations of *Ls* (S2 Table). Final *Ls* concentrations in assay cups ranged from 0.0005 to 51,200 ppb (µg/L); this range was established during the study by adding concentrations as needed to capture the full range of responses. (S2 Table). Control cups without larvicide were established in triplicate per sample site and run in parallel. Mortality was assessed 48 hours after treatment. In keeping with the methods described in [13,14], and [22] moribund larvae (larvae unable to swim or maintain a position at the surface of the water) were classified as dead. Control mortality was averaged (mean) and used to correct observed mortality using Abbott’s formula [23].

Probit regression (R studio, version 4.3.3) [24] was used to calculate LC_50_, LC_90_ and 95% confidence interval values for each population using the package ‘ecotox’ [25–27]. As recommended in [28], a Pearson’s χ² goodness-of-fit test was used to assess whether the observed bioassay mortality data were consistent with the expectations of a probit model. Only statistically significant data sets were included. In 3 sample populations, a significant probit curve could not be fit due to insufficient larvae to add more replicates and/or concentrations. Resistance ratios were calculated by dividing the LC_50_ or LC_90_ of each sampled field population by the respective value for the susceptible strain (COL). Populations were categorized by *Ls* resistance intensity as follows: susceptible (RR_50_ < 5), moderate resistance (RR_50_ ≥ 5 and < 10), or high resistance (RR_50_ ≥ 10), consistent with WHO guidance for *Aedes* mosquitoes [29].

To establish diagnostic doses for S-methoprene resistance testing, we generated dose–response data using a susceptible *Cx. pipiens* laboratory strain (COL). Diagnostic doses were defined as 10× (1.84 ppb) and 100× (18.43 ppb) the susceptible LC₅₀, (0.18 ppb; 95% CI 0.11–0.29) corresponding to thresholds for low or moderate resistance (RR_50_ < 10), high (RR₅₀ = 10–100) and extreme (RR₅₀ > 100) resistance, respectively, as defined in previous publications [17,29]. Late fourth-instar COL larvae were exposed to 20 concentrations between 0.001 ppb and 1000 ppb of technical-grade S-methoprene (Item 33375, Sigma-Aldrich, St. Louis MO, USA), prepared in analytical-grade acetone (Item 270725, Sigma-Aldrich), using 3–15 replicates per concentration. The test range and methods closely mirrored those used for the *Ls* assays described above, including matched bioassay conditions and statistical procedures. As S-methoprene is a juvenile hormone analog that disrupts emergence, mortality was defined as failure to emerge successfully and included dead larvae, dead pupae, or incompletely emerged adults [13,14,17]. Probit analysis of corrected mortality data, followed by a Pearson’s χ² goodness-of-fit test, yielded the susceptible LC₅₀ value used to calculate the diagnostic doses.

Each field-collected population was exposed to at least three and up to ten replicates per diagnostic dose of S-methoprene, depending on larval availability. Control cups (no larvicide) were included in triplicate for each group and run in parallel. Mortality in controls was averaged (mean) and used to correct observed mortality using Abbott’s formula [23]. Mortality was assessed and recorded after all larvae had either emerged successfully or died. For each diagnostic dose, mean corrected mortality and standard error of the mean were calculated. Resistance intensity for each sample population was categorized as follows: Susceptible/Low Resistance: > 50% mortality at both RR_50_ 10 and RR_50_ 100; High Resistance: < 50% mortality at RR_50_ 10 and >50% at RR_50_ 100; Extreme Resistance: < 50% mortality at both doses.

### Estimating the probability of *Ls* resistance

Since 2016, NSMAD has recorded all larvicide treatments to stormwater catch basins using FieldSeeker GIS (Frontier Precision, Bismarck, ND, USA), a geographic information system that enables storage and retrieval of detailed treatment data. For sample locations with a history of *Ls* application—specifically, operational zones A01, A07, A09, B01, B08, B19, C02, C11, C13, C15, C18, C21, C24 —the total number of *Ls* treatments applied to catch basins (the primary use site for *Ls*) between 2016 and 2024 was divided by the total number of catch basins mapped in the GIS database for each sample location to calculate the average number of *Ls* treatments per catch basin for each location. Because catch basins are spatially fixed and received repeated treatments at consistent doses (20 g of Vectolex granules per treatment (Valent BioSciences, Libertyville, IL, USA)) over time, they represent discrete units of cumulative exposure, allowing us to directly correlate treatment intensity with observed resistance outcomes. The number of *Ls* treatments per year varied between 0 and 3 for each operational zone. The remaining 19 sample locations had no documented history of *Ls* exposure yielding an average of 0 treatments per catch basin.

To test the hypothesis that resistance to S-methoprene is associated with resistance to *Ls*, independent of *Ls* exposure history, we used Fisher’s Exact Test to evaluate the association between these two binary traits across mosquito populations. Since prior laboratory work observed *Ls* cross resistance when levels of S-methoprene resistance reached RR_50_ = 168 [19], this analysis aimed to examine whether the presence of “high” (RR_50_ ≥ 10) to “extreme” (RR_50_ ≥ 100) S-methoprene resistance (as defined in [17]) increased the likelihood of observing any *Ls* resistance (RR_50_ ≥ 5). Since S-methoprene resistance was assessed using a diagnostic dose assay (rather than a dose response assay) and this assay can only produce a binary yes/no result at a predefined threshold, there was no opportunity to generate an RR_50_ for S-methoprene or to evaluate a quantitative relationship with *Ls* RR_50_. Fisher’s Exact Test was chosen because the data included small expected cell counts, which violate the assumptions of the chi-square test. The analysis was performed using GraphPad Prism version 10.4.1 (GraphPad Software, Boston, MA, USA).

The probability of *Ls* resistance based on the average number of *Ls* treatments to catch basins within each sample zone between 2016 and 2024 was evaluated using a logistical regression model. Separate models were created for RR_50_ and RR_90_ data sets. The response variable was the presence or absence of *Ls* resistance (RR_50_ or RR_90_ ≥ 5). The two explanatory variables considered were the average number of *Ls* treatments per catch basin in each site with a *Ls* treatment history and the binary presence or absence of S-methoprene resistance at RR_50 _≥ 10. The collection site was explored as a random effect. Model selection was completed with backwards selection, and Akaike information criterion (AIC) and log likelihood for nested models were considered [30]. Model fits were evaluated for selected models. All logistical regression analyses were completed in R studio, version 4.3.3 [24] with packages ‘lme4’ [31], ‘performance’ [32] and ‘AICcmodavg’ [33]. All logistical regression graphs were created with GraphPad Prism version 10.4.1 (Graph Pad Software, Boston, MA, USA).

## Results

**Fig 2:**
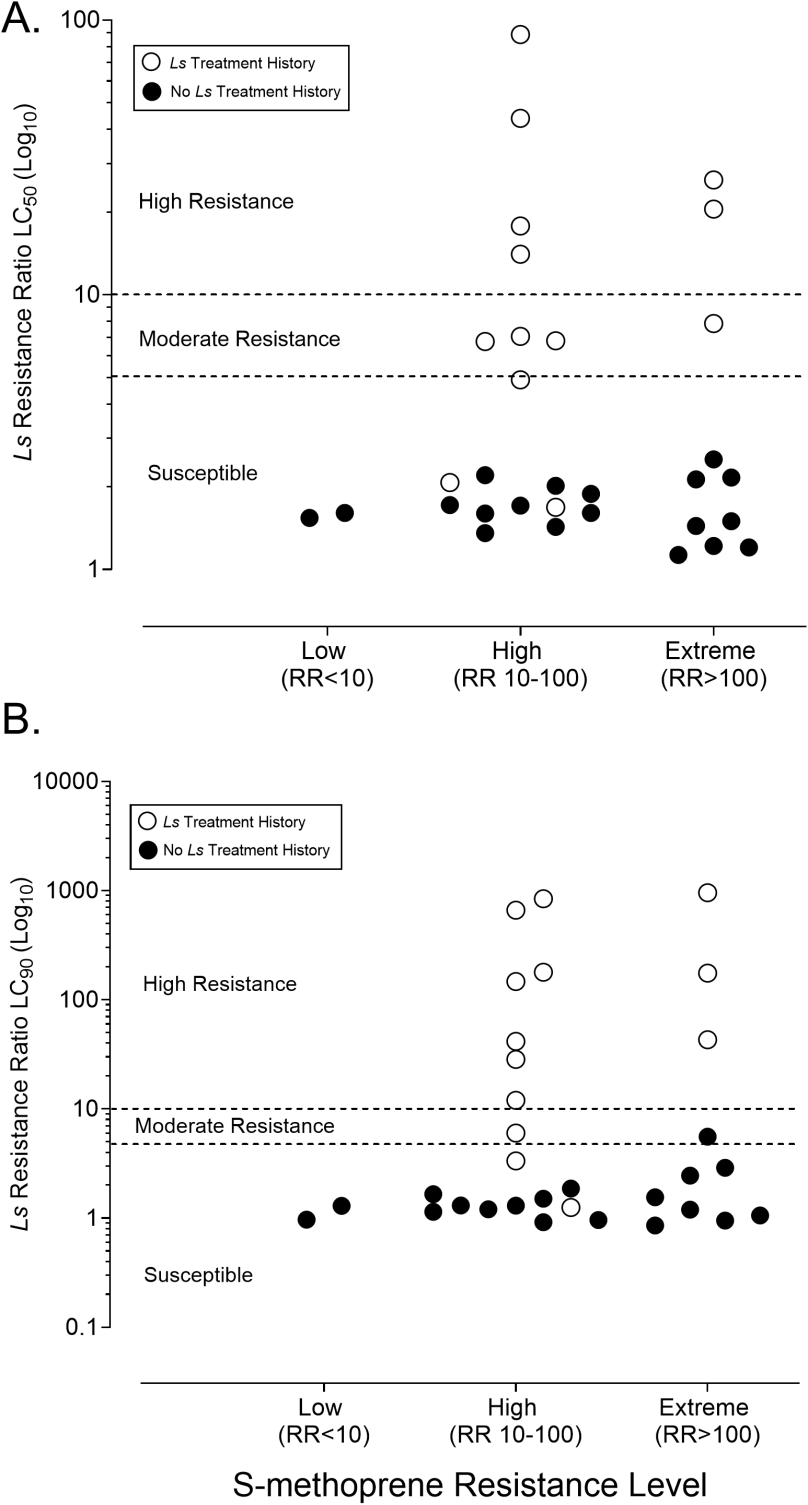
Comparison of resistance categories of S-methoprene with *Ls* resistance ratios in 32 field-collected *Cx. pipiens* populations. **(A)** S-methoprene resistance categories vs. LC_50_ *Ls* resistance ratios (log_10_ scale) and **(b)** S-methoprene resistance categories vs. LC_90_ values (log_10_ scale) **(B)**. White circles indicate populations from sites with *Ls* treatment history, where black circles indicate populations without *Ls* treatment history.

Diagnostic-dose bioassays demonstrated at least a high level of resistance to S-methoprene (RR_50_ > 10 but <100) as defined in [29] in almost 94% of populations where it has been used extensively (30 out of 32 populations) with 34% of populations (11 out of 32) demonstrating an “extreme” level of resistance (RR_50_ > 100) as defined in [17] (Fig 2; S3 Table). Only two populations (BAR and HOF) with a S-methoprene treatment history showed a level of resistance to S-methoprene below RR_50_ 10, the threshold for “high resistance” utilized in this study (S3 Table). The susceptible strain (COL) exhibited similar LC_50_ and LC_90_ values to previous work [17,28], indicating that this strain remains a good reference for comparison (S4 Table).

Field collected *Cx. pipiens* from the 32 sample locations demonstrate that “moderate” to “high” *Ls* resistance (RR_50_ ≥ 5) was present in 77% (10 out of 13 populations) with a *Ls* treatment history (Table 1; Fig 2A). Only 23% (3 populations) with a *Ls* treatment history remained fully susceptible at RR_50_ (Table 1; Fig 2A). Of note, one of these populations (A01) demonstrated an RR_50_ of 4.91 (95% CI 3.95–6.07), just below the threshold for “moderate resistance” (Table 1). Resistance ratios at the LC_50_ ranged as high as 88.58 (95% CI 62.66–124.16; Table 1). Resistance ratios at the LC_90_ were often much higher than those based on the LC_50_ in populations with *Ls* treatment history, ranging from 1.25 (95% CI 0.84–2.27) to a maximum of 955.76 (95% CI 294.76–5615.10; Table 1).

**Table 1:** Summary of dose response data, resistance ratios, and statistical results for field-collected larval *Cx. pipiens* exposed to *Ls.*

District	*Ls* History?	Collection site	N(n)^a^	Slope ± s.e.m.	P-value^b^	LC_50_ (µg/L) (95% CI^c^)	LC_90_ (µg/L) (95% CI^c^)	RR_50_^d^ (95% CI^c^)	RR_90_^e^ (95% CI^c^)
**NWMAD**	N	COL	86 (1982)	1.69 ± 0.08	<1.00 × 10^-100^	9.98 (0.97–4.67 × 10^5^)	55.17 (12.49–6.60 × 10^33^)	–	–
	N	BAR	48 (1068)	2.32 ± 0.15	4.98 × 10^−6^	14.96 (12.09–18.22)	53.47 (41.71–74.05)	1.54 (1.25–1.88)	0.97 (0.76–1.34)
	N	HOF	49 (1110)	1.94 ± 0.11	1.28 × 10^-17^	15.62 (11.61–20.46)	71.39 (50.48–116.70)	1.61 (1.20–2.11)	1.29 (0.91–2.12)
	N	AHC	56 (1252)	1.61 ± 0.08	7.40 × 10^-40^	13.19 (8.77–19.54)	82.88 (50.19–175.77)	1.36 (0.90–2.01)	1.50 (0.91–3.19)
	N	AHN	44 (990)	2.51 ± 0.17	5.39 × 10^−3^	15.61 (13.13–18.32)	50.64 (41.22–66.05)	1.61 (1.35–1.89)	0.92 (0.75–1.19)
	N	DPN	58 (1277)	1.32 ± 0.07	<1.00 × 10^-100^	14.57 (6.58–30.44)	134.98 (56.69–823.19)	1.49 (0.68–3.13)	2.45 (1.03–14.92)
	N	DPS	44 (988)	1.90 ± 0.12	6.35 × 10^−7^	14.00 (10.83–17.64)	65.96 (48.87–99.08)	1.44 (1.11–1.82)	1.19 (0.89–1.79)
	N	WHE	65 (1425)	1.09 ± 0.05	1.60 × 10^−5^	20.68 (15.01–27.66)	307.26 (214.51–474.98)	2.13 (1.55–2.85)	5.57 (3.89–8.61)
	N	PKR	36 (794)	2.35 ± 0.16	2.24 × 10^-23^	24.46 (17.39–34.61)	85.78 (55.79–180.95)	2.52 (1.79–3.56)	1.55 (1.01–3.28)
	N	11S	36 (810)	2.41 ± 0.18	5.33 × 10^-29^	15.54 (9.92–22.66)	52.90 (34.33–114.83)	1.60 (1.02–2.33)	0.96 (0.62–2.08)
	N	17S	64 (1427)	1.95 ± 0.10	3.70 × 10^−2^	13.89 (11.99–15.97)	63.02 (51.98–79.34)	1.43 (1.24–1.64)	1.14 (0.94–1.44)
	N	24S	44 (1024)	2.16 ± 0.14	1.08 × 10^−2^	18.31 (15.35–21.61)	71.78 (57.12–96.34)	1.88 (1.58–2.22)	1.30 (1.04–1.75)
	N	12P	49 (1066)	2.13 ± 0.14	3.32 × 10^−2^	16.59 (13.85–19.69)	66.44 (53.03–88.10)	1.71 (1.43–2.02)	1.20 (0.96–1.59)
	N	21P	67 (1484)	1.78 ± 0.09	2.27 × 10^−2^	19.59 (16.73 – 22.88)	103.07 (82.89–133.67)	2.02 (1.72–2.36)	1.87 (1.03–1.75)
	N	15M	51 (1119)	2.02 ± 0.12	1.03 × 10^−4^	16.68 (13.63–20.28)	71.94 (55.03–101.74)	1.72 (1.40–2.09)	1.30 (0.99–1.84)
	N	17W	36 (782)	2.04 ± 0.15	2.79 × 10^−4^	21.43 (16.95–27.03)	91.27 (65.68–145.76)	2.21 (1.75–2.78)	1.65 (1.19–2.64)
**DVMAD**	N	BRO	44 (999)	2.02 ± 0.13	2.11 × 10^−7^	10.98 (8.44–13.91)	47.23 (35.22–70.05)	1.13 (0.87–1.43)	0.86 (0.63–1.27)
	N	LAG	43 (938)	1.84 ± 0.12	1.29 × 10^−2^	11.69 (9.478–14.16)	58.30 (45.46–79.89)	1.20 (0.98–1.46)	1.06 (0.82–1.45)
	N	MAY	39 (880)	1.46 ± 0.09	3.63 × 10^-28^	21.01 (11.81–35.82)	159.51 (83.21–474.50)	2.16 (1.22–3.69)	2.89 (1.51–8.59)
	N	OPS	47 (1057)	1.99 ± 0.13	3.23 × 10^−2^	11.84 (9.84–13.97)	52.33 (41.98–68.38)	1.22 (1.01–1.44)	0.95 (0.77–1.24)
**NSMAD**	Y	A01	45 (950)	1.52 ± 0.09	1.41 × 10^−2^	47.66 (38.39–58.99)	331.37 (243.56–487.37)	4.91 (3.95–6.07)	6.01 (4.41–8.83)
	Y	A07	53 (1134)	0.94 ± 0.05	3.53 × 10^-32^	68.61 (38.91–115.46)	1570.05 (756.65–4.67 × 10^3^)	7.06 (4.04–11.89)	28.45 (13.71–84.79)
	Y	A09	45 (1009)	2.05 ± 0.14	3.81 × 10^-20^	16.36 (10.84–23.22)	69.09 (46.23–125.36)	1.68 (1.12–2.39)	1.25 (0.84–2.27)
	Y	B01	69 (1514)	0.83 ± 0.04	1.46 × 10^-20^	66.11 (42.09–100.83)	2.29 × 10^3^ (1.27 × 10^3^ – 4.89 × 10^3^)	6.81 (4.33–10.38)	41.54 (23.14–88.54)
	Y	B08	44 (955)	0.55 ± 0.04	3.52 × 10^−8^	254.44 (137.85–487.47)	5.27 × 10^4^ (1.62 × 10^4^ – 3.09 × 10^5^)	26.19 (14.19–50.19)	955.76 (294.76–5615.10)
	Y	B19	33 (694)	1.33 ± 0.09	2.09 × 10^−3^	20.15 (14.60–27.25)	184.21 (121.37–322.75)	2.07 (1.50–2.81)	3.34 (2.19–5.85)
	Y	C02	69 (1510)	1.28 ± 0.06	7.03 × 10^-82^	65.72 (38.88–107.38)	662.99 (352.12–1.77 × 10^3^)	6.76 (4.00–11.06)	12.02 (6.38–32.04)
	Y	C11	51 (1042)	0.74 ± 0.04	5.49 × 10^−2^	860.55 (608.59–1.21 × 10^3^)	4.66 × 10^4^ (2.76 × 10^4^ – 8.82 × 10^4^)	88.58 (62.66–124.16)	844.10 (501.01–1599.37)
	Y	C13	73 (1628)	0.66 ± 0.03	5.13 × 10^-11^	426.41 (286.43–634.76)	3.65 × 10^4^ (1.89 × 10^4^ – 8.38 × 10^4^)	43.89 (29.49–65.36)	661.35 (342.89–1518.43)
	Y	C15	70 (1511)	0.77 ± 0.03	1.114 × 10^-41^	173.07 (98.23–301.21)	8.12 × 10^3^ (3.67 × 10^3^ – 2.43 × 10^4^)	17.82 (10.11–31.01)	147.19 (66.56–439.51)
	Y	C18	80 (1736)	0.69 ± 0.03	2.76 × 10^-64^	136.47 (75.43–250.83)	9.87 × 10^3^ (3.71 × 10^3^ – 4.27 × 10^4^)	14.05 (7.77–25.83)	178.82 (67.17–773.64)
	Y	C21	92 (1995)	0.86 ± 0.03	<1.00 × 10^-100^	76.40 (40.71–142.69)	2.38 × 10^3^ (1.01 × 10^3^ – 8.45 × 10^3^)	7.86 (4.19–14.69)	43.10 (18.27–153.12)
	Y	C24	39 (794)	0.76 ± 0.05	3.46 × 10^−2^	199.31 (129.49–297.52)	9.69 × 10^3^ (5.45 × 10^3^ – 2.02 × 10^4^)	20.52 (13.33–30.63)	175.65 (98.82–365.99)

COL = susceptible colony *Cx. pipiens*. ^a^Number of replicates tested (number of mosquitoes tested). ^b^P-value for Pearson’s χ^2^ goodness-of-fit test. ^c^95% Confidence Interval. ^d^LC_50_ resistance ratio. ^e^LC_90_ resistance ratio.

Conversely, there was no *Ls* resistance at RR_50_ (RR_50_ = 1.1–2.5) observed in the remaining 19 populations that had no history of *Ls* exposure (Table 1; Fig 2A). *Ls* treatment history and *Ls* resistance at the RR_50_ exhibited near perfect separation in the observed data; all *Ls* resistant populations had a documented history of *Ls* exposure, and no resistance was detected in populations lacking such exposure (Fig 2A). This pattern remained true for both the “high” and “extreme” S-methoprene resistant populations (Fig 2A). Out of the 13 populations with a *Ls* treatment history, 6 exhibited “high” resistance, 4 exhibited “moderate” resistance, and 3 exhibited “susceptible/low” resistance (one “low” sample location exhibited an RR_50_ to *Ls* of 4.91). The *Ls* RR_90_ values of these populations remained similar to the RR_50_ and ranged from 0.86–5.57 (Table 1). Of note, one population (WHE) was not considered resistant to *Ls* at the LC_50_ dose but exhibited a higher resistance ratio at the LC_90_ dose (RR_90_ = 5.57), though this value is extremely close to the cutoff for this tier of resistance (RR = 5) (Table 1). Out of 13 populations with a *Ls* treatment history, 10 exhibited “high” resistance, 1 “moderate” resistance and 2 exhibited “susceptible/low” resistance at the RR_90_ (Fig 2B).

Based on published observations of cross-resistance between S-methoprene and *Ls* in artificial selection experiments [19], we hypothesized that populations exhibiting S-methoprene resistance at RR_50_ ≥ 10, despite no history of *Ls* treatment, would also show resistance to *Ls* at RR_50_ ≥ 5. However, Fisher’s Exact Test found no evidence of an association between S-methoprene and *Ls* resistance across populations (two-tailed p = 0.9999). Of the 32 populations sampled, 20 were resistant to S-methoprene only at RR_50_ > 10, 0 were resistant to *Ls* only at RR_50_ ≥ 5, 10 exhibited multiple resistance to both larvicides, and only 2 populations were susceptible to both.

**Fig 3:**
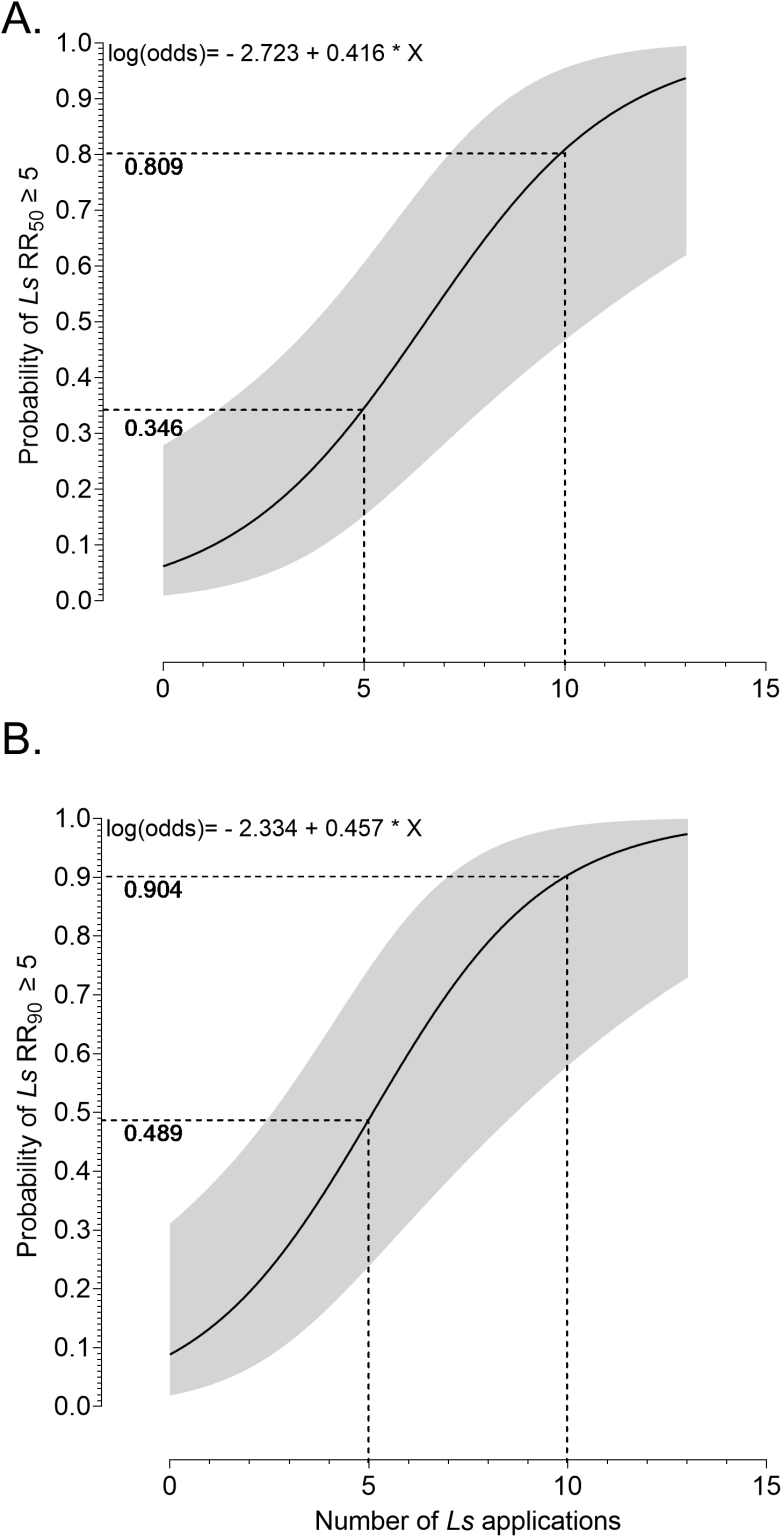
The probability of development of *Ls* resistance at. **(A)** RR_50 _≥ 5 and **(B)** RR_90 _≥ 5 in *Cx. pipiens* populations based on the average number of *Ls* treatments to a catch basin within the operational zone/sample location.

A logistic regression was used to estimate the probability of resistance to *Ls* as a function of the average number of *Ls* applications to catch basins from 2016 to 2024. As shown in Fig 3A and 3B, the probability of resistance increases sharply with the number of *Ls* treatments (p = 0.002; Table 2). After 10 applications, the probability of resistance, defined here as RR_50_ ≥ 5, exceeds 80%. The final models for both RR_50_ and RR_90_ included the average number of *Ls* treatments as the sole explanatory variable (Table 2; S5 Table). An odds ratio of 1.51 for RR_50_ indicates that each additional *Ls* treatment increases the odds of resistance by approximately 51% (Table 2). Operationally, this corresponds to a probability of resistance of 0.346 after 5 treatments, and 0.809 after 10 treatments (Fig 3). For RR_90_, the odds ratio was slightly higher at 1.58, yielding a probability of resistance of 0.904 after 10 treatments (Table 2, Fig 3).

**Table 2:** Final logistical regression chosen for likelihood of *Ls* resistance associated with *Ls* use.

	*Ls* Resistance at RR_50_			*Ls* Resistance at RR_90_		
*Predictors*	*Odds Ratios*	*95% CI*	*p*	*Odds Ratios*	*95% CI*	*p*
(Intercept)	0.07	0.01–0.28	**0.002**	0.10	0.01–0.35	**0.003**
Number of *Ls* treatments	1.51	1.22–2.08	**0.002**	1.58	1.25–2.17	**0.001**
Observations	32			Observations		32
R^2^ Tjur	0.499			R^2^ Tjur		0.580

## Discussion

*Lysinibacillus sphaericus* resistance observed in this study was completely unrelated to the presence of S-methoprene resistance in our field collected *Cx. pipiens*—a result that contrasts sharply with prior laboratory selection experiments (Fig 2) [19]. We examined the relationship between S-methoprene resistance (defined as RR_50_ ≥ 10) and *Ls* resistance (RR_50_ ≥ 5) and found no evidence of an association between the two (p = 0.9999). This result supports the conclusion that *Ls* resistance in these regional *Cx. pipiens* populations is not occurring independently of *Ls* exposure and is not driven by underlying resistance to S-methoprene. In our dataset, nearly all populations were resistant to S-methoprene, but only those with a history of *Ls* treatment exhibited *Ls* resistance. All populations without *Ls* exposure remained susceptible at the RR_50_, regardless of their S-methoprene resistance status.

In this study, we detected moderate to high resistance to *Ls*, as defined by the WHO [29], with a maximum RR_50_ of 88.6 and RR_90_ of 844.1 (Table 1). Most sample locations with a history of *Ls* treatment to stormwater catch basins exhibited some level of resistance (Table 1; Fig 1). In the Chicago, Illinois region, *Ls* has been primarily used as a stormwater catch basin treatment where the average density of catch basins can exceed 300 basins/Km^2^ leading to a high volume of larvicidal treatments. Because of this density of larval mosquito habitat, we also assessed the relationship between *Ls* treatment history and the probability of developing *Ls* resistance. The results of this analysis indicate that the estimated probability of *Ls* resistance increases rapidly; after just 5 treatments, the probability of resistance at RR_50_ exceeded 30%, and after just 10 treatments, it exceeded 80% (Fig 3). The odds ratio of our logistical regression indicates that each additional *Ls* treatment is associated with over a 50% increase in the odds of resistance forming (Table 2). It is important to note that *Ls* applications occurred between 0 and 3 times per year for a period of 8 years and that other active ingredients were used between and within years of *Ls* use, suggesting that *Ls* resistance may have been more severe if product rotations had not occurred.

Such development of resistance to *Ls* has been noted in the literature in numerous published examples. In California, use of *Ls* for only 1.5 yrs led to resistance and control failure [13]. In Utah, 12 years of use of *Ls* in catch basins also led to near complete resistance as well as described control failures [14]. In the example from Thailand, only 5 treatments to polluted surface waters led to a complete loss of field effectiveness [22]. Numerous other examples of rapid resistance development to *Ls,* independent of use pattern, in *Cx. pipiens* complex mosquitoes have been documented (reviewed in [7]). The *Ls* resistance we measured developed within 8 years (2016–2024) and within a maximum of 12 treatments. In most sample locations that exhibited *Ls* resistance, *Ls* exposure occurred in only 5 out of 8 years with product rotations occurring during the intervening years. In one sample location, C24, only 4 treatments with *Ls* resulted in a RR_50_ of 20.5 despite rotation with 3 other active ingredients (Table 1). In stark contrast, mosquito populations with no exposure to *Ls* remained completely susceptible at the LC_50_ to this active ingredient (Table 1). Taken together with previously published evidence, these results clearly demonstrate that resistance to *Ls* in *Cx. pipiens* populations can develop very rapidly. MCOs intending to use *Ls* to control *Culex* spp. must account for these limitations in their long-term planning and resistance management strategies.

Despite the identification of moderate to high resistance in our sample locations, far higher resistance ratios have been documented in *Cx. pipiens* populations from across the United States. In Chico, California, RR_50_ and RR_90_ values exceeded 537.0 and 9048.5, respectively; levels associated with control failure [13]. Similarly, in Salt Lake City, Utah, observed control failures led to measurements of an RR_50_ of 20,780.0 and an RR_90_ of 23,926.9 in field populations [14]. Globally, *Ls* resistance in *Cx. pipiens* complex mosquitoes has been reported in France, India, Brazil, China, Thailand, and Tunisia [reviewed in 7]. In Thailand, one population exhibited an RR_50_ of 28,100 to a formulated *Ls* product [22]. Efforts to associate resistance levels with residual activity of *Ls* in catch basins were not formally assessed in this study and further research is needed in this regard.

Much of the existing literature describing cross-resistance has been based on laboratory colonies, which may have experienced genetic bottlenecks at some stage, potentially leading to unique laboratory artifacts from genetic drift or founder effects that are not widely applicable [34]. Field-collected mosquitoes, by contrast, likely possess greater genetic diversity, including alternative and independent resistance mechanisms and therefore, a broader range of evolutionary pathways to resistance development. It is most likely that the resistance mechanisms present in the field-collected populations in this study differ from those observed in laboratory-selected colonies; a pattern which can explain the absence of observed cross-resistance in our data. It is worth noting there was one S-methoprene resistance population (WHE) with no known *Ls* treatment history that exhibited an RR_90_ of 5.57 (Table 1). It is possible that this single population, resistant at the LC_90_ (but susceptible at the LC_50_) is an indication of the cross-resistant relationship observed in laboratory studies. However, when this single observation is compared against the 18 other sample locations with no *Ls* treatment history and no *Ls* resistance, it suggests that any such cross-resistance relationship, if it exists, would be rare.

Although the absence of cross-resistance between S-methoprene and *Ls* in field populations of *Cx. pipiens* is an encouraging finding, it is important to note that 10 of the sampled populations still exhibited independent resistance to both larvicides. To our knowledge, this represents the first documented case of simultaneous resistance to S-methoprene and *Ls* in field-collected *Cx. pipiens*. Surprisingly, only two of the 32 tested populations (BAR and HOF) were fully susceptible to both S-methoprene and *Ls*. The methodology used in this study allowed for both larvicides to be tested on the same mosquito populations, collected at the same time, providing direct evidence of concurrent resistance. By design, the experimental approach employed here controlled for temporal variation, eliminating potential confounding factors from changes in resistance over time or the migration of mosquitoes in and out of the sample area. The detection of dual resistance underscores the need to account for the development of *Ls* resistance when developing rotational strategies for larvicide resistance management, as current recommendations do not consider the speed at which resistance can emerge, the potential for multiple resistance, or the specific selection pressures associated with different treatment patterns.

In the face of widespread insecticide resistance and a limited range of resistance management tools, MCOs must balance the need to suppress local vector mosquito populations with the imperative to manage and delay resistance. Unfortunately, the suite of available resistance management methods is extremely limited. Common resistance management strategies used in agriculture include integrated pest management (IPM) [35], pesticide mosaic applications, the establishment of genetic refuges [36], product rotation using different active ingredients, switching to alternative chemistries, and combining (i.e., “stacking”) active ingredients with distinct modes of action [11,37,38]. Except for product rotation [5] and the use of an IPM framework [5,6], many of the resistance management strategies suggested for agricultural settings have limited to no adoption in MCOs or in integrated vector management frameworks. Our observations are congruent with a variety of genetic models which have demonstrated a very limited utility of rotations in slowing the evolution of resistance development [39–41]. Our results further suggest that a recommendation of product rotation alone, without considering rotation timing, rotational order, or the specific characteristics or interactions of the insecticides used, was insufficient to prevent the development of resistance to *Ls*.

Because *Ls* activity is highly specific to larval mosquitoes and demonstrates little to no activity in other insects, it is unlikely that the selective pressure for *Ls* resistance originates from alternate sources such as commercial pest control or private residential applications. Most S-methoprene applications to catch basins are similarly restricted to mosquito control organizations in this region. In accordance with our previous work [17], it is most likely that MCOs in the Chicago, IL region have inadvertently driven resistance to *both* S-methoprene and *Ls* despite employing IPM/IMM methods and rotational schemes. Nonetheless, it is not outside the realm of possibility that urban runoff containing other pesticides and other chemicals in stormwater catch-basins may be inducing or enhancing resistance or cross resistance to *Ls* or S-methoprene.

In conclusion, the results described here demonstrate that *Ls* resistance developed in Cx. pipiens in most locations in the Chicago metropolitan area where *Ls* applications were made, and this resistance was independent of pre-existing S-methoprene resistance. We also demonstrated that resistance to *Ls* developed in locations where it was used resulting in resistance to multiple active ingredients. Together, these results underscore the urgent need for further research into effective resistance management strategies, as current rotation-based recommendations lack the specificity or detail required for successful implementation in the field.

## Supporting information

S1 Table GPS coordinates of egg collection sites. (DOCX)

S2 Table Number of replicates per collection site and concentration of *Ls.* Susceptible colony mosquitoes denoted by COL. Untreated controls for mortality correction are listed as “control”.
(DOCX)

S3 Table Susceptibility of field-collected larval *Cx. pipiens* to two diagnostic doses of technical grade S-methoprene. (DOCX)

S4 Table Determination of S-methoprene diagnostic dose of susceptible laboratory *Cx. pipiens.* (DOCX)

S5 Table Model selection table for probability of *LS* resistance. Models within 2 AIC were considered equal. Final model selection is bold. Reporting includes the terms included in the regression, the distribution (negative binomial), df (K), AIC, Delta AIC, AIC weight, and Log Likelihood.
(DOCX)

S6 File Raw bioassay mortality data. (XLSX)
