# Supplementary material for: Dual S-methoprene and Lysinibacillus sphaericus larvicide use leads to multiple independent, and not cross-resistance in Culex pipiens
Source: PLoS One. 2025 Sep 29;20(9):e0332621. doi: 10.1371/journal.pone.0332621 (PMC12478903; doi:10.1371/journal.pone.0332621)
Supplement: S1 Table — (DOCX) [file pone.0332621.s001.docx]

**S1 Table.** **GPS coordinates of egg collection sites.**

| Collection Site | GPS coordinates of ovitraps | |
| --- | --- | --- |
| BAR | 42°06'39"N 88°09'40"W  42°07'14"N 88°10'30"W | 42°06'58"N 88°10'34"W  42°06'41"N 88°10'33"W |
| HOF | 42°02'53"N 88°13'18"W  42°03'21"N 88°13'12"W | 42°03'23"N 88°14'17"W  42°02'53"N 88°14'33"W |
| AHC | 42°04'37"N 87°59'34"W  42°04'49"N 88°00'09"W | 42°05'08"N 88°00'10"W |
| AHN | 42°06'25"N 88°00'17"W  42°06'16"N 87°59'17"W | 42°06'20"N 87°59'28"W  42°05'57"N 87°59'47"W |
| DPS | 42°02'02"N 87°55'56"W  42°02'00"N 87°55'35"W | 42°01'36"N 87°55'38"W |
| DPN | 42°03'20"N 87°54'45"W  42°03'50"N 87°54'47"W | 42°03'48"N 87°54'30"W  42°03'48"N 87°54'23"W |
| WHE | 42°06'59"N 87°55'53"W  42°06'36"N 87°55'57"W | 42°07'01"N 87°56'44"W  42°06'50"N 87°56'46"W |
| PKR | 42°00'24"N 87°49'34"W  41°59'51"N 87°49'55"W | 41°59'50"N 87°50'07"W  42°00'27"N 87°50'27"W |
| 11S | 42°03'47"N 88°02'57"W  42°03'25"N 88°03'16"W | 42°03'35"N 88°03'56"W  42°03'08"N 88°04'04"W |
| 17S | 42°02'50"N 88°06'54"W  42°02'41"N 88°06'46"W | 42°02'34"N 88°06'42"W  42°02'16"N 88°06'43"W |
| 24S | 42°01'50"N 88°02'19"W  42°01'42"N 88°02'35"W | 42°01'35"N 88°02'06"W  42°01'29"N 88°02'13"W |
| 12P | 42°07'32"N 88°01'04"W  42°07'34"N 88°01'08"W | 42°07'44"N 88°00'52"W |
| 21P | 42°06'34"N 88°04'31"W  42°06'33"N 88°04'03"W | 42°06'11"N 88°04'08"W  42°05'51"N 88°04'22"W |
| 15M | 42°02'34"N 87°50'56"W  42°02'59"N 87°51'28"W | 42°02'58"N 87°51'47"W  42°02'27"N 87°51'54"W |
| 17W | 42°06'49"N 87°58'01"W  42°07'20"N 87°58'23"W | 42°07'13"N 87°58'14"W |
| LAG | 41°48'37"N 87°51'58"W  41°48'46"N 87°51'51"W | 41°48'53"N 87°51'51"W  41°49'09"N 87°51'53"W |
| OPS | 41°52'16"N 87°47'22"W  41°52'15"N 87°47'12"W | 41°52'15"N 87°47'04"W  41°52'15"N 87°46'57"W |
| MAY | 41°51'38"N 87°49'56"W  41°51'37"N 87°49'42"W | 41°51'42"N 87°49'43"W |
| BRO | 41°51'41"N 87°51'48"W | 41°51'38"N 87°51'54"W |
| A01 | 42°08'36"N 87°49'46"W  42°08'50"N 87°49'47"W | 42°09'04"N 87°50'42"W  42°08'59"N 87°50'50"W |
| A07 | 42°07'06"N 87°44'35"W  42°07'18"N 87°44'51"W | 42°07'30"N 87°45'05"W |
| A09 | 42°07'24"N 87°48'45"W  42°07'39"N 87°49'26"W | 42°07'30"N 87°49'35"W  42°07'24"N 87°49'33"W |
| B01 | 42°05'50"N 87°50'06"W  42°05'53"N 87°49'29"W | 42°05'43"N 87°50'01"W |
| B08 | 42°05'44"N 87°43'31"W  42°05'25"N 87°43'16"W | 42°05'09"N 87°42'56"W  42°04'54"N 87°42'45"W |
| B19 | 42°03'15"N 87°42'23"W  42°03'21"N 87°42'06"W | 42°03'38"N 87°42'40"W  42°03'50"N 87°43'27"W |
| C02 | 42°03'10"N 87°47'45"W  42°03'12"N 87°47'08"W | 42°03'03"N 87°47'36"W |
| C11 | 42°01'41"N 87°45'35"W | 42°02'24"N 87°45'32"W |
| C13 | 42°02'07"N 87°43'19"W  42°02'16"N 87°42'36"W | 42°01'48"N 87°42'37"W |
| C15 | 42°01'52"N 87°41'08"W  42°01'57"N 87°40'48"W | 42°01'55"N 87°40'32"W  42°01'55"N 87°40'13"W |
| C18 | 42°00'47"N 87°44'20"W  42°01'13"N 87°44'17"W | 42°01'15"N 87°44'44"W |
| C21 | 42°00'37"N 87°47'50"W  42°00'30"N 87°47'46"W | 42°00'21"N 87°47'35"W  42°00'10"N 87°47'24"W |
| C24 | 41°59'54"N 87°43'35"W  41°59'55"N 87°43'07"W | 42°00'16"N 87°43'04"W |
