# Supplementary material for: Dual S-methoprene and Lysinibacillus sphaericus larvicide use leads to multiple independent, and not cross-resistance in Culex pipiens
Source: PLoS One. 2025 Sep 29;20(9):e0332621. doi: 10.1371/journal.pone.0332621 (PMC12478903; doi:10.1371/journal.pone.0332621)
Supplement: S2 Table — Susceptible colony mosquitoes denoted by COL. Untreated controls for mortality correction are listed as “control”. (DOCX) [file pone.0332621.s002.docx]

**S2 Table: Number of replicates per collection site and concentration of *Ls***. Susceptible colony mosquitoes denoted by COL. Untreated controls for mortality correction are listed as “control.”
