# Supplementary material for: Dual S-methoprene and Lysinibacillus sphaericus larvicide use leads to multiple independent, and not cross-resistance in Culex pipiens
Source: PLoS One. 2025 Sep 29;20(9):e0332621. doi: 10.1371/journal.pone.0332621 (PMC12478903; doi:10.1371/journal.pone.0332621)
Supplement: S3 Table — (DOCX) [file pone.0332621.s003.docx]

**S3 Table. Susceptibility of field-collected larval *Cx. pipiens* to two diagnostic doses of technical grade s-methoprene.**

| Collection Site | N(n) ^a^ Diagnostic Dose 1 (RR 10) | N(n) ^a^ Diagnostic Dose 2 (RR 100) | Mean mortality at Diagnostic Dose 1 ± SE | Mean mortality at Diagnostic Dose 2 ± SE | Resistance Intensity |
| --- | --- | --- | --- | --- | --- |
| BAR | 8 (179) | 7 (164) | 0.64 ± 0.02 | 0.96 ± 0.01 | Low |
| HOF | 9 (200) | 9 (195) | 0.51 ± 0.02 | 0.94 ± 0.01 | Low |
| AHC | 5 (102) | 5 (107) | 0.24 ± 0.04 | 0.69 ± 0.04 | High |
| AHN | 6 (133) | 6 (128) | 0.13 ± 0.02 | 0.76 ± 0.02 | High |
| DPN | 7 (150) | 7 (148) | 0.25 ± 0.03 | 0.46 ± 0.03 | Extreme |
| DPS | 6 (141) | 6 (133) | 0.14 ± 0.02 | 0.47 ± 0.04 | Extreme |
| WHE | 10 (214) | 9 (186) | 0.22 ± 0.01 | 0.45 ± 0.01 | Extreme |
| PKR | 5 (106) | 4 (80) | 0.21 ± 0.04 | 0.44 ± 0.04 | Extreme |
| 11S | 6 (124) | 6 (124) | 0.42 ± 0.03 | 0.84 ± 0.01 | High |
| 17S | 7 (149) | 6 (128) | 0.41 ± 0.03 | 0.82 ± 0.01 | High |
| 24S | 10 (220) | 10 (229) | 0.41 ± 0.02 | 0.82 ± 0.02 | High |
| 12P | 7 (148) | 7 (145) | 0.16 ± 0.01 | 0.69 ± 0.01 | High |
| 21P | 8 (177) | 8 (170) | 0.24 ± 0.02 | 0.65 ± 0.03 | High |
| 15M | 10 (220) | 10 (223) | 0.25 ± 0.02 | 0.67 ± 0.01 | High |
| 17W | 3 (59) | 3 (61) | 0.23 ± 0.04 | 0.64 ± 0.02 | High |
| BRO | 9 (205) | 10 (221) | 0.05 ± 0.01 | 0.27 ± 0.01 | Extreme |
| LAG | 8 (181) | 8 (179) | 0.14 ± 0.01 | 0.21 ± 0.01 | Extreme |
| MAY | 6 (131) | 6 (129) | 0.23 ± 0.02 | 0.25 ± 0.01 | Extreme |
| OKP | 10 (222) | 10 (220) | 0.13 ± 0.01 | 0.47 ± 0.01 | Extreme |
| A01 | 5 (108) | 5 (111) | 0.29 ± 0.05 | 0.69 ± 0.04 | High |
| A07 | 10 (209) | 10 (202) | 0.21 ± 0.01 | 0.57 ± 0.02 | High |
| A09 | 10 (222) | 10 (212) | 0.18 ± 0.01 | 0.70 ± 0.01 | High |
| B01 | 4 (80) | 3 (61) | 0.46 ± 0.02 | 0.73 ± 0.01 | High |
| B08 | 10 (213) | 10 (204) | 0.24 ± 0.01 | 0.49 ± 0.01 | Extreme |
| B19 | 6 (127) | 6 (123) | 0.33 ± 0.03 | 0.73 ± 0.05 | High |
| C02 | 10 (210) | 10 (213) | 0.33 ± 0.02 | 0.64 ± 0.02 | High |
| C11 | 5 (101) | 5 (100) | 0.21 ± 0.02 | 0.75 ± 0.03 | High |
| C13 | 10 (214) | 10 (218) | 0.19 ± 0.01 | 0.55 ± 0.02 | High |
| C15 | 10 (204) | 10 (199) | 0.33 ± 0.01 | 0.62 ± 0.01 | High |
| C18 | 10 (207) | 10 (205) | 0.25 ± 0.02 | 0.56 ± 0.01 | High |
| C21 | 4 (81) | 4 (82) | 0.03 ± 0.01 | 0.27 ± 0.01 | Extreme |
| C24 | 5 (103) | 5 (99) | 0.08 ± 0.01 | 0.42 ± 0.04 | Extreme |

^a^Number of replicates tested (number of mosquitoes tested)
