# Supplementary material for: Dual S-methoprene and Lysinibacillus sphaericus larvicide use leads to multiple independent, and not cross-resistance in Culex pipiens
Source: PLoS One. 2025 Sep 29;20(9):e0332621. doi: 10.1371/journal.pone.0332621 (PMC12478903; doi:10.1371/journal.pone.0332621)
Supplement: S4 Table — (DOCX) [file pone.0332621.s004.docx]

**S4 Table. Determination of s-methoprene diagnostic dose of susceptible laboratory *Cx. pipiens*.**

| Collection Site | N(n)^a^ | Slope ± s.e.m. | P-value^b^ | LC_50_ (µg/L)  (95% CI^c^) | LC_90_ (µg/L)  (95% CI^c^) | Diagnostic Dose 1: 10X LC_50_ Dose (µg/L) | Diagnostic Dose 2: 100X LC_50_ Dose (µg/L) |
| --- | --- | --- | --- | --- | --- | --- | --- |
| COL | 141 (3100) | 0.59 ± 0.02 | 2.51e-52 | 0.18 (0.11 – 0.29) | 25.32 (14.37 – 50.16) | 1.84 | 18.43 |

^a^Number of replicates tested (number of mosquitoes tested)

^b^P-value for Pearson’s χ^2^ goodness-of-fit test

^c^95% Confidence Interval
