# Supplementary material for: Dual S-methoprene and Lysinibacillus sphaericus larvicide use leads to multiple independent, and not cross-resistance in Culex pipiens
Source: PLoS One. 2025 Sep 29;20(9):e0332621. doi: 10.1371/journal.pone.0332621 (PMC12478903; doi:10.1371/journal.pone.0332621)
Supplement: S5 Table — Models within 2 AIC were considered equal. Final model selection is bold. Reporting includes the terms included in the regression, the distribution (negative binomial), df (K), AIC, Delta AIC, AIC weight, and Log Likelihood. (DOCX) [file pone.0332621.s005.docx]

**S5 Table. Model selection table for probability of *LS* resistance.** Models within 2 AIC were considered equal. Final model selection is bold. Reporting includes the terms included in the regression, the distribution (negative binomial), df (K), AIC, Delta AIC, AIC weight, and Log Likelihood.

| **Regression terms** | **PDF** | **K** | **AICc** | **Delta AIC** | **AICcWt** | **LL** |
| --- | --- | --- | --- | --- | --- | --- |
| **Number of LS treatments** | **nb** | **2** | **27.57** | **0** | **5.79e-1** | **-11.58** |
| Number of *LS* treatments + s-methoprene resistance | nb | 3 | 29.74 | 2.17 | 1.96e-1 | -11.44 |
| Number of *LS* treatments + (1\|Site) | nb | 3 | 30.02 | 2.44 | 1.71e-1 | -11.58 |
| Number of *LS* treatments + s-methoprene resistance + (1\|Site) | nb | 4 | 32.37 | 4.79 | 5.28e-2 | -11.44 |
| s-methoprene resistance | nb | 2 | 42.60 | 15.03 | 3.16e-4 | -19.09 |
| s-methoprene resistance + (1\|Site) | nb | 3 | 45.05 | 17.47 | 9.31e-5 | -19.09 |
